# Tumor HPV Status, Level of Regulatory T Cells and Macrophage Infiltration Predict up to 20-Year Non-Disease-Specific Survival in Oropharynx Squamous Cell Carcinoma Patients

**DOI:** 10.3390/biomedicines10102484

**Published:** 2022-10-05

**Authors:** Hilde Haave, Borghild Ljokjel, Helene Lybak, Svein E. Moe, Jan E. Berge, Olav K. Vintermyr, Lars Helgeland, Hans J. Aarstad

**Affiliations:** 1 Department of Otolaryngology/Head and Neck Surgery, Haukeland University Hospital, 5020 Bergen, Norway; 2 Department of Pathology, Haukeland University Hospital, 5020 Bergen, Norway; 3 Department of Clinical Medicine, Faculty of Medicine, University of Bergen, 5020 Bergen, Norway

**Keywords:** cancer, immunology, oropharynx

## Abstract

Oropharynx squamous cell carcinoma (OPSCC) is of special interest because human papilloma virus (HPV) and/or smoking cause this disease. Influxes of inflammatory cells into such tumors are known to vary with prognoses. Aims: To study whether the density of tumor-infiltrating T lymphocytes and tumor-infiltrating macrophages predicted general 20-year overall survival (OS), as well as OS with only disease-specific survival (DSS) patients included. Methods: Biopsies from patients treated for OPSCC (*n* = 180) were stained by immunohistochemistry and the tumor cell macrophage (CD68), pan T lymphocytes (CD3), and regulatory T lymphocytes (Foxp3) densities were determined. The HE-determined percentage of matured tumor cells and the rate of invasion were calculated, and stromal desmoplasia were performed. Tumor HPV presence was studied by PCR. Twenty-year OS and five-year DSS patients were determined. Results: Tumor HPV status strongly predicted survival. High tumor infiltration of CD3, Foxp3 and CD68-positive cells predicted better twenty-year OS, with and without HPV stratification. Foxp3 and CD68 levels predicted OS, and 20-year among DSS patients, primarily among HPV(+) patients. Tumor HE-derived variables did not predict such survival. Conclusions: Tumor HPV status, level of Foxp3 tumor-infiltrating lymphocytes and CD68 tumor-infiltrating macrophages predicted up to 20-year OS of both all patients and disease-specific survived patients.

## 1. Introduction

Head and neck cancer (HNC) is the sixth most common cancer in the world, if non-melanoma skin cancer patients are excluded [1]. HNC kills about 50% of those who are diagnosed worldwide [2]. In the Western world, one third of the patients diagnosed with head neck squamous cell carcinoma (HNSCC) suffer disease-specific death (DSD) within five years [3]. Typically, HNSCC most often recur within three years, corresponding to DSD within five years [4]. The overall survival (OS) beyond five years in patients treated for HNSCC as the index disease, however, rapidly declines with time [4]. The main aim of this investigation was to study this process and its association to the immune system more closely.

HNCs are a heterogenic group of diseases with, to some extent, different causes, treatment and prognosis, with site as an important discriminator [5]. We have previously studied, in particular, oropharyngeal SCC (OPSCC), and therefore find it pertinent presently to use such patients. An advantage with this disease origin is that one may include investigation of different profiles between HPV-caused SCC versus OPSCC generated by tobacco and alcohol consumption [6]. These two different causes generate OPSCC with different tumor immune infiltration, histological malignancy degree and prognosis [7]. Few investigators have investigated in detail the impact of these two causes of the disease on long-term prognosis, and this consequently forms the main aim of this investigation.

When measured from basic HE slide levels of nuclear polymorphism, invasion, degree of stromal desmoplasia and lymphocyte infiltration relates to disease-specific survival (DSS) in OPSCC patients [8]. We aimed to study to what extent this is the case regarding long-term survival.

Evidence suggests links between both general and specific immunity regarding cancer, which both defend against [9] and support [10] tumor growth. This seems to be especially important with virally caused malignancies [11]. Influxes of various inflammatory cells into tumors are known to signal prognosis of malignant tumors [12].The number of both tumor-infiltrating lymphocytes (TILs) [13] and tumor-associated macrophages (TAMs) [14] may be tied to prognosis of cancer. HNSCC cancer may serve as an example of worse prognosis following an increasing number of TAMs in general [15]. On the other hand, such TIL and macrophage responses have been associated with a better prognosis in OPSCC patients [16]. Thus, the presence of inflammation in OPSCC behaves partially different than in other carcinomas, further suggesting that immunity in carcinomas originating from these sites is of high interest.

CD3 is a general marker identifying T lymphocytes [17]. T lymphocytes includes Regulatory T (Treg) cells co-regulating immune responses. Tregs represent 5–10% of peripheral CD4-positive T lymphocytes [18]. “Repressive forkhead or winged-helix family transcription factor” (Foxp3) is one important marker of regulatory T cells [19]. It has been shown that levels of Foxp3-positive lymphocytes in tumors predict DSS of OPSCC patients [7,16]. We therefore find it relevant to study Foxp3 lymphocyte infiltration in tumors from OPSCC patients regarding long-term prognosis.

The mononuclear phagocyte (MNP) system includes monocytes, macrophages and some dendritic cells [20]. MNPs in tissue differentiate primarily to either M1 or M2 macrophages with pro-inflammatory or anti-inflammatory properties, respectively [21]. M1 macrophages produce pro-inflammatory cytokines, such as interleukin-1β (IL-1β), IL-6 and TNF-α; [21] and M2 macrophages are inhibitory [22,23]. In particular, a large body of evidence supports the connection between IL-6 and cancer development [24]. In a review paper, the IL-6 serum level at diagnosis was significantly correlated to survival in 82/101 series comprising 9917 out of 11,583 patients with 23 different cancer types [24], including HNSCC [25] patients. We [26,27] and others [6] have also shown an association between monocyte tumor reactivity and HNSCC prognosis. We therefore aimed to study the level of macrophages as measured by CD68-positive cell infiltration in the studied OPSCC tumors in relation to long-term prognosis.

The TNM stage is known to be important in predicting oropharyngeal DSS [4]. Its influence regarding long-term survival is not well known. Therefore, this formed an adjustment of this investigation. Furthermore, around the year 2000, we changed treatment from primarily RT to surgery and postoperative RT. We have shown that these two treatments had similar DSS [28]. We therefore found it pertinent to adjust only by TNM stage and not by treatment when studying long-term survival.

We aimed to study 20-year OS in OPSCC patients, with and without disease-specific-death (DSD) patients subtracted and stratified by tumor HPV presence, dependent on the activation level of TILs measured by Foxp3-positive cell numbers. The level of tumor-infiltrating macrophages (TIM) measured by CD68-positive cells was also studied. Furthermore, we studied the long-term prognosis secondary to tumor histological degree of stromal desmoplasia, general lymphocyte infiltration, nuclear polymorphism and degree of tumor invasion.

## 2. Materials and Methods

### 2.1. Patients Included in the Study

From 1 January 1992, all patients diagnosed with head and neck cancer (HNC) at the regional Haukeland University Hospital (HUH), Bergen, Department of Otolaryngology/Head & Neck Surgery, Norway, were registered in an HNC registry. The hospital draws patients from about one million individuals.

All HN squamous cell carcinoma (HNSCC) patients were subjected to standard diagnostic work-up, including clinical examination, CT/MRI scans of the primary tumor, neck, thorax, and liver, and ultra-sonography of the neck, including fine needle aspiration cytology if indicated. Endoscopic examination under general anesthesia was performed if possible. The patients with OPSCC diagnosed in the period from 1992 until 2008 were extracted. The five-year disease-specific survival (DSS) and 20-year overall survival (OS) rates were established. The treatments of the patients have been reported elsewhere [28]. In total, 170 patients were included.

### 2.2. Immunohistochemistry

Tumor tissue samples from the OPSCC were fixed in buffered formalin and embedded in paraffin. Sections were cut at 4 μm. The following antibodies were used in this study: anti CD68 (clone KP1, Dako/Agilent, Santa Clara, CA, USA, dilution 1:3000), anti CD3 (rabbit polyclonal, DAKO/Agilent, dilution 1:100) and anti Foxp3 (clone 259D/C7, BD Pharmingen, San Jose, CA 95131, USA, dilution 1:20). Antigen retrieval and staining were performed fully automated in a Ventana Benchmark Ultra according to the manufacturer’s instructions.

### 2.3. Scoring of IHC

The material was evaluated concomitantly in a two-headed microscope by the primary investigator (BL) and an experienced pathologist (LH). Scoring was done by counting the number of positive cells per HPF at 630× magnification in a Zeiss Axio microscope (Carl Zeiss, Jena, Germany), calculating the mean number of positive cells in five randomly chosen neighboring fields. If the counts in two fields exceeded 200 cells, calculation of the mean number was based on those fields. The tumor epithelium was evaluated. All sections were evaluated blindly. Foxp3 status was successfull obtained from 166, CD3 status from 164 and CD68 status from 153 patients, respectively.

### 2.4. Histological Evaluation

One pathologist (OKV) and an experienced ENT surgeon (HH) scored the histology grading as reported previously [8]. If possible, the grading was obtained blindly and the scoring was done at an invasive edge. The morphologic features were given scores from 1 to 4. Low numbers were considered to represent the least aggressive tumors and high numbers the most aggressive tumors, according to the original histological malignancy grading system developed by Kristensen et al. [29]. Mature cells were scored as a percentage of mature cancer cells and scored most (≥75%) to scarce (≤25%). Pattern of invasion was scored as (1) “pushing” with well-delineated, infiltrating borders; (2) “infiltrating” with solid cords, bands and/or strands; (3) “small groups or cords of infiltrating cells”; (4) “marked and widespread cellular dissociation in small groups and/or single cell”. Tumor host inflammatory response was assessed as the degree of inflammatory cells (lymphocytes, plasma cells and macrophages) around tumor cell islands and scored 1–4 depending on the presence of a marked, moderate, slight or close to no inflammatory response. Tumor stromal desmoplasia was scored as the degree of fibroblast response around tumor cell islands and scored 1–4, i.e., close to none, slight, moderate or marked.

### 2.5. DNA Isolation

All tumor samples were reviewed by an expert in pathology (OKV), and representative tissue samples were selected. DNA was extracted from formalin-fixed, paraffin-embedded (FFPE) specimens.

### 2.6. HPV DNA Detection

We have previously published this method in detail [30]. Among the HPV tumor-positive, one patient was HPV-18-positive, one patient was HPV-33-positive and one patient was HPV-35-positive, the rest HPV-16 positive. All other information from the patients was gathered and entered into the database without knowledge of the HPV status.

### 2.7. Calculating Standard Mortality

The standardized mortality ratio (SMR) represents the relative mortality of the study population compared with the expected mortality in a reference population. The expected number of deaths was calculated as the total number of person years at risk for each sex-specific age group, with 5-year intervals, multiplied by the corresponding death rate in the Norwegian population for the same groups.

### 2.8. Statistics

The statistical program package PASW was employed (Ver. 26; SPSS Inc. Chicago, IL, USA) for the analysis as indicated. Values of *p* < 0.05 were considered statistically significant results. All *p*-values reported represent two-sided tests. The associations between the possible prognostic variables with survival were determined using a Kaplan–Meier estimator (log rank option) and/or Cox proportional hazards regression models. The Cox regression survivals are reported as relative risk (RR) with confidence intervals.

## 3. Results

### 3.1. Clinical Parameters Sorted by Tumor HPV Presence, Age of Patient at Diagnosis, Tumor Site and TNM Stage

The present cohort (*n* = 170) represents a subgroup of the entire group of patients diagnosed in Western Norway with OPSCC (*n* = 280) in the period from 1992 until 30 June 2008. Table 1 shows the age, gender and TNM stage by HPV tumor presence: 76 had tumors that were HPV(−) and 92 had tumors that were HPV(+). Two patients had an unknown HPV status. The mean age among the HPV(−) was 61.1 ± 11.0 years, versus 58.6 ± 10.5 years among the HPV(+) (Table 1).

### 3.2. Twenty-Year Survival Dependent on Tumor HPV Status

**Figure 1:**
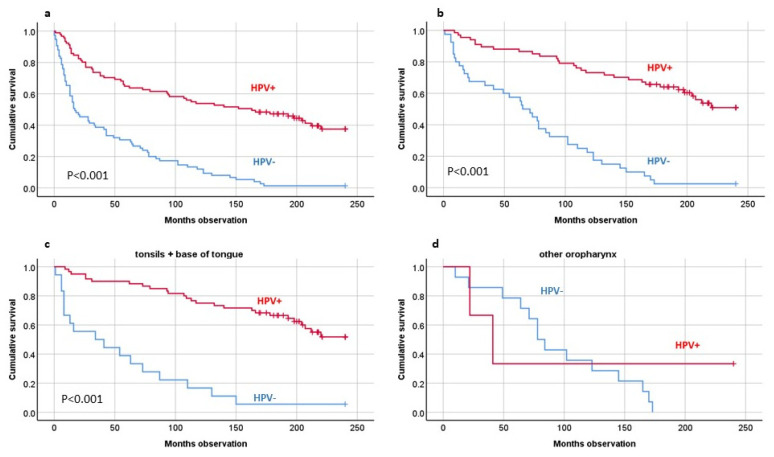
Twenty-year overall survival, including all patients (**a**), disease-specific-survival patients (**b**), patients with tumors originated in the tonsils or base of the tongue (**c**), or elsewhere in the oropharynx (**d**). HPV analyses by PCR analyses. Statistics by Kaplan–Meier analyses.

Among all included patients, 133 died throughout the observation period, compared to 21 expected from standard Norwegian mortality rate, i.e., a relative risk (RR) of 6.2 and confidence interval (CI) of 5.2–7.4 (Table 2). Among the HPV(+) patients, 56 died, compared to 14 expected (RR = 4.1 with CI 3.2–5.3) and among the HPV(−), 75 died, compared to eight expected (RR = 9.8 with CI 7.8–12.3) (Table 2). Only among the HPV(+) patients did age predict 20-year overall survival (*p* < 0.001). TNM stage predicted OS only among the HPV(−) patients, i.e., T (*p* = 0.042), N (*p* = 0.016) and M (*p* = 0.001), respectively (Table 1). Positive HPV tumor infection status strongly predicted better 20-year OS (*p* < 0.001) (Figure 1a). This was also the case if the disease-specific-death (DSD) patients were subtracted (*p* < 0.001) (Figure 1b).

If the patients were sorted by HPV tumor status and by site, HPV tumor status predicted long-term survival among patients with tumors of tonsillar or BOT origin (*p* < 0.001). The same was the case with only the DSS patients included (*p* < 0.001) (Figure 1c,d).

### 3.3. Twenty-Year Survival by Kaplan–Meier Analyses, Studying TIL CD3, FoxP3 or TIM CD68 Positivity

**Figure 2:**
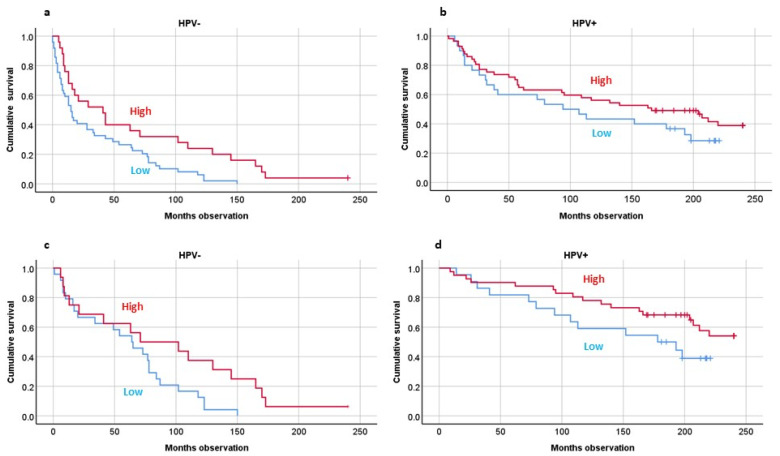
Twenty-year overall survival studying density of tumor-infiltrating T lymphocytes (CD3) by HPV tumor presence, including all patients (**a**,**b**) or including disease-specific-survival patients (**c**,**d**). HPV analyses by PCR analyses. CD3 analyses by immunohistochemistry. Statistics by Kaplan–Meier analyses: Overall survival combined: *p* = 0.005. Disease-specific survival combined: *p* = 0.01.

We found that patients with a high number of CD3-positive TILs had better twenty-year OS than those with low numbers (*p* < 0.001). This also applied following sorting by HPV tumor status (*p* = 0.005) (Figure 2a,b). If only the DSS patients were included, the survival prediction was still valid (*p* = 0.01) (Figure 2c,d).

**Figure 3:**
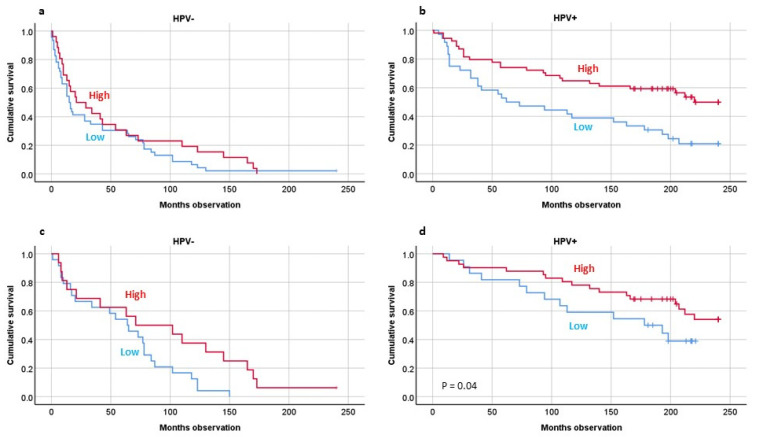
Twenty-year overall survival studying Foxp3-positive (T regulatory) cells by HPV tumor presence including all patients (**a**,**b**) or including disease-specific-survival patients (**c**,**d**). HPV analyses by PCR analyses. Foxp3 analyses by immunohistochemistry. Statistics by Kaplan–Meier analyses: Overall survival combined: *p* = 0.005; Disease-specific survival combined (**c**,**d**) (HPV(+) only): *p* = 0.04.

For Foxp3-positive TILs, we showed that high numbers of TILs were tied to better twenty-year OS by Kaplan–Meier analyses (*p* < 0.001). This was also the case stratified by tumor HPV presence (*p* < 0.01) (Figure 3a,b). If the patients were sorted into two groups based on HPV status, including DSS patients only, this was significant concerning the HPV tumor-positive individuals (*p* = 0.04) (Figure 3c,d).

**Figure 4:**
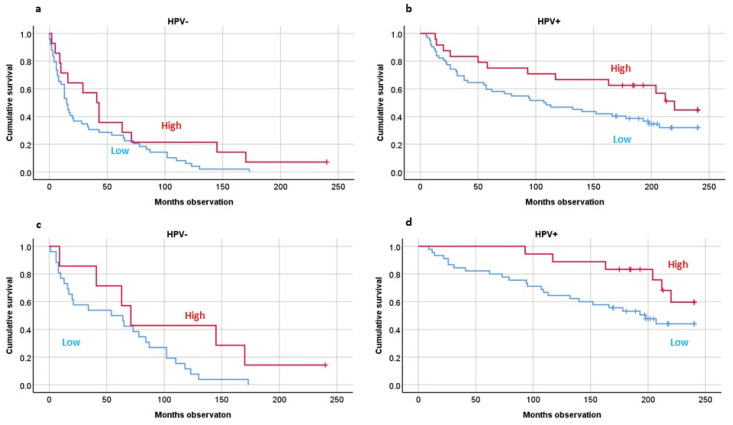
Twenty-years overall survival studying CD68-positive cells (macrophages) by HPV tumor presence, including all patients (**a**,**b**) or including disease-specific-survival patients (**c**,**d**). HPV analyses by PCR analyses. CD68 analyses by immunohistochemistry. Statistics by Kaplan–Meier analyses: Overall survival combined: *p* = 0.021; Disease-specific survival combined: *p* = 0.015.

Concerning CD68 expression, patients with high such numbers also had better 20-year OS than the patients with low TIMs (*p* = 0.012) (Figure 4a,b). Including only DSS patients, the level of CD68 expression still predicted OS (*p* = 0.018) (Figure 4c,d).

### 3.4. Twenty-Year Survival by Kaplan–Meier Analyses Studying Tumor Cell Fraction of Mature Cells (Nuclear Polymorphism), Pattern of Invasion, Tumor Stromal Desmoplasia and Tumor Host Inflammatory Response

**Figure 5:**
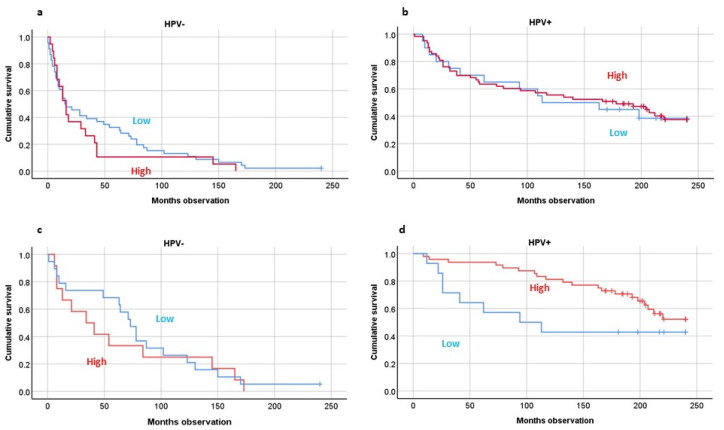
Twenty-year overall survival studying nuclear polymorphism level by HPV tumor presence including all patients (**a**,**b**) or studying lymphocyte density including disease-specific-survival patients (**c**,**d**). HPV analyses by PCR analyses. Nuclear polymorphism and lymphocyte density analyses by HE slides. Survival curves by Kaplan–Meier analyses. Significance: None.

High nuclear polymorphism signaled better twenty-year overall survival, directly analyzed (*p* < 0.001). If only DSS patients were included, nuclear polymorphism still predicted survival (*p* < 0.001). If the patients were also stratified by tumor HPV tumor status, the rate of nuclear polymorphism did not predict survival (Figure 5a,b).

As with nuclear polymorphism, pattern of high invasion (*p* = 0.007), marked level of desmoplasia (*p* < 0.001), as well as low level of inflammation (*p* < 0.001), predicted worse OS. If studied with DSS patients only, the survival prediction was lost regarding invasion and desmoplasia (results not shown), but not rate of inflammation (*p* = 0.001). If the patients additionally were stratified by HPV tumor status, none of these variables retained general prognostic value when measured by twenty-year survival (Figure 5c,d). Regarding the inflammatory response, among the HPV(+) patients, the survival prediction up to 175 months was maintained (Figure 5d).

### 3.5. Twenty-Year Survival by Cox Multivariate Regression Analyses by TIL FoxP3, TIL CD3 and TIM CD68, Whether HE-Histology Generated Levels of Tumor Desmoplasia, Inflammation, Nuclear Ploidity, Invasion, Age of the Patient and T Stage, and Whether HPV Tumor Positivity

Cox stepwise regression twenty-year multivariate survival analyses were performed, including age at diagnosis, gender, TNM stage of tumor, HPV tumor status and smoking history as the first block, additionally with one of the following variables as the second block: CD3 TIL cells, FoxP3 TIL cells, tumor CD68 cells, HE-inflammatory cells, HE-desmoplasia, HE-nuclear polymorphism or HE-invasion (Table 3). Studying 20-year overall survival, Foxp3 (*p* = 0.008), CD3 (*p* = 0.039) and CD68 (*p* = 0.011) levels were significant predictors. A trend was observed regarding rate of desmoplasia (*p* = 0.084) and inflammation (*p* = 0.06) (Table 3). If including only DSS patients, predictions were still observed regarding CD3 TIL cells (*p* = 0.028), FoxP3 TIL cells (*p* = 0.017) and CD68 tumor cells (*p* = 0.005) (Table 3).

## 4. Discussion

Among OPSCC patients, we have presently shown that HPV tumor positivity signals excellent host up to twenty-year overall survival (OS). HPV tumor status strongly predicted both long-term OS and OS with disease-specific death (DSD) excluded, but only among patients with tonsillar or BOT origin. A strong immune response to the cancer, as measured by high tumor influx of CD68(+), CD3(+) or Foxp3(+) cells, predicts better long-term overall survival, especially Foxp3(+) in HPV(+) tumor patients. This also applied when disease-specific-death (DSD) individuals were removed from the cohort.

HPV(+) OPSCC cancers have better disease-specific survival (DSS) than smoking and alcohol-related OPSCC [31]. The present study shows that HPV+ OPSCC patients have much better long-term OS and OS with DSD individuals subtracted than HPV(−) OPSCC patients do. The reason remains unknown. We have tested this by adjusting for smoking history, but the complete explanation was not determined. However, we observed only three deaths among the non-smoking HPV(+) patients following cure of the cancer disease, which is as naturally suspected. With a higher number of patients included, such a conclusion could have been more reliable. In addition, we have previously studied in OPSCC patients the non-OPSCC survival dependent on the level of periodontitis at diagnosis, and shown that the level of periodontitis predicts non-OPSCC smoking-adjusted survival [32]. Such a prediction may be secondary to passive smoking [32] and possibly sheds light on other explanatory variables, i.e., passive smoking and inaccurately reported smoking history. An additional explanatory variable is alcohol intake [33], but reliable data concerning this are not available. Other explanations may also apply.

HPV(+) tumor patients do not have prognostic advantage across all sites compared to HPV(−) patients; i.e., BOT and tonsillar origin only merits better prognosis [34]. This seems to be important in regard to long-term prognosis in DSS too [35]. Other HNSCC sites, such as the larynx, may also be host to HPV-positive cancer, but here no DSS-changed prognosis can be drawn from HPV status [36]. No one has offered any explanation as to the prognostic advantage of BOT/tonsillar sites, but this is an extremely interesting area of further investigation.

Level of lymphocyte infiltration as measured by HE histology is a marker for DSS in OPSCC patients [37]. We [7] and others [16] have shown for both HPV(−) and HPV(+) HNSCC patients better DSS for high levels of TIL CD3 [16]. Presently, we have shown that favorable long-term OS is also attached to high tumor levels of TIL T cells. If the DSD patients were removed and HPV tumor stratification applied, histology-based long-term survival prediction was not retained.

Regulatory T cells are important for immunological homeostasis and peripheral self-tolerance [18]. Tregs interact with B and T lymphocytes, monocytes, macrophages, dendritic cells and mast cells [38]. These interactions are primarily hypothesized to cause suppression of autoimmune disease [18]. High levels of FoxP3 lymphocytes are linked to worse cancer prognosis, e.g., colorectal, melanomas and lung carcinomas [39]. On the other hand, high levels of FoxP3-positive lymphocytes may signal better cancer prognoses, e.g., breast, prostate and gastric cancers [39]. In the case of Foxp3-positive cells, we [7] have shown that better DSS was associated with a high TIL Foxp3+ level in OPSCC patients. The present analyses confirmed this regarding twenty-year OS and even if the results were stratified by HPV tumor content and the DSD patients removed from the analyses. This was also true relative to age, gender, TNM stage and smoking history, studied by Cox multivariate analysis, but primarily shown among the HPV(+) tumor patients. What can be stated presently is that Tregs presumably interact more closely with immune processes important to survival within patients with HPV-generated tumors compared to patients with smoking and alcohol-generated OPSCC tumors. Treg density, presumably coupled to specific immunity, also signals better long-term survival. Why so motivates further research.

Presently, we have also studied the level of TAMs, i.e., CD68(+) cells, in relation to long-term prognosis. Even if DSD patients were removed and the patients were stratified by HPV tumor presence, the TAM levels remained predictive. This is in line with much general information that supports that the natural immune system functional status, in particular IL-6 levels, is related to general survival in cancer patients [24]. Such findings are, in general, however, not extended to non-DSS or drawn from intra-tumor material. Thus, it is of interest to note that both macrophage and Foxp3(+) TIL density located within the tumor is seemingly associated to general non-OPSCC survival twenty years later. The hypothesis that the immune system within tumors is affected by the general condition of the host should be further studied.

We have previously shown that several histology-derived variables, i.e., percentage of mature cells, rate of invasion and stromal desmoplasia, predict prognosis in OPSCC patients [39]. This is also found regarding long term OS, but not when DSD patients were removed from the cohort. Thus, these changes in the cancer cells bear limited relation to the general non-OPSCC survival expectations.

This study is one of several that encourages further studies about immune-based treatment of OPSCC. A specific immune response directed against tumor cells is presumably the fundamentals both under the present Foxp3 results and the effects of modern immunotherapy [40]. Immunotherapy is a therapy with potentially profound efficiency, but not all patients experience this, and this underlines the importance of choosing the correct patients as candidates for immunotherapy [41]. It is possible to use information gathered along many lines in order to predict patient response to immunotherapy [42], but simpler approaches are easier to use everywhere. To study the TIL Foxp3 level in HNSCC patients eligible for immunotherapy to determine treatment result could be part of a future simple suggestion in order to better aim such treatment. The present result also encourages us to study whether macrophage-aimed therapy, e.g., IL-6 blockage, could be used as an OPSCC treatment.

With additionally included patients, this study could have had more conclusions. Furthermore, a multi-center study would be preferable. On the other hand, the patients originate from one cohort, including most of the eligible patients from one geographical area, and the study is therefore presumably not biased. Each patient was closely monitored and thus the basic clinical results are accurate. The immunohistochemical staining was done as a single procedure, eliminating drift in this method over time.

## 5. Conclusions

In conclusion, HPV(+) patients have much better prognosis than HPV(−) patients. We have furthermore shown that high levels of tumor CD3(+), CD68(+) cells and TIL FoxP3 predict better twenty-year OS, both overall and among non-OPSCC-related deaths. The FoxP3 prediction is best found among HPV(+) tumors. These survival predictions were, to some extent, independent of TNM stages, age and gender. The present results encourage further studies regarding the immune system and related treatment in OPSCC patients.

## Figures and Tables

**Table 1:** Clinical patient characteristics and twenty-year overall survival, including at-diagnosis patient age, gender and TNM stage by HPV status.

	Tumor HPV(−)		Tumor HPV(+)	
		Sign. 20 Y OS		Sign. 20 Y OS
Age		n.s.		<0.001
Years Mean ± SD	62.1 ± 11.0		58.6 ± 10.5	
Gender (N)		n.s.		n.s.
Males	63		65	
Females	13		27	
T stage		0.042		n.s.
1	12		14	
2	20		31	
3	24		31	
4	18		16	
N stage		0.016		n.s.
0	29		18	
1	14		4	
2	25		65	
3	5		5	
M stage		0.001		n.a.
0	68		92	
1	3		0	
Smoking		n.s.		0.064
0	3		11	
1	1		10	
2	2		12	
3	15		29	
4	48		26	
Total patients included	76		92	

Smoking score: 0: Never smoked. 1: Smoked fewer than 10 packs/year. 2: Probably fewer than 10 packs/yaer. 3: Probably more than 10 packs/year. 4: More than 10 packs/year. n.s. = Not significant. n.a. = Not applicable.

**Table 2:** Mortality of included patients compared to expected number according to age and sex-matched standardized Norwegian national mortality.

Patients	Number of Patients	Number of Deaths	Expected Deaths	RR	Confidence Interval
From diagnosis					
All	168	133	21	6.2	5.2–7.4
HPV(+)	91	56	14	4.1	3.2–5.3
HPV(−)	75	75	8	9.8	7.8–12.3
Patients with survival more than 5 years following diagnosis					
All	84	49	19	2.6	2.0–3.5
HPV(+)	59	24	12	1.9	1.3–2.9
HPV(−)	23	23	6	3.8	2.5–5.7
HPV(+) Non-smokers	7	2	1.3	1.5	0.4–6.2

**Table 3:** Cox multivariate regression twenty-year overall survival with and without disease-specific five-year survival analysis excluded. Included were at-diagnosis gender, age, TNM stage, HPV tumor status, tobacco consumption history, as well as tumo- infiltrating levels of FoxP3, CD3, CD68 lymphocyte/macrophages (TIL/TIM), whether HE-histology generated levels of desmoplasia, inflammation and nuclear ploidity, or invasion measured binomially.

	20-Year OS				20-Year DSS Only			
	Sign.	RR	95% CI for RR		Sign.	RR	95% CI for RR	
			Lower	Upper			Lower	Upper
Block I								
Gender	0.531	0.843	0.494	1.438	0.651	0.857	0.441	1.668
Age of patient	0.000	1.06	1.035	1.083	0.000	1.077	1.041	1.113
T-stage	0.073	1.20	0.983	1.471	0.234	1.194	0.892	1.599
N-stage	0.006	1.36	1.095	1.698	0.289	1.174	0.873	1.578
M-stage	0.016	7.18	1.437	35.832				
HPV	0.000	0.37	0.235	0.588	0.002	0.364	0.193	0.686
Tobacco history	0.002	1.35	1.114	1.625	0.007	1.432	1.105	1.856
Block I plus levels of TIL CD3								
TIL CD3	0.039	0.651	0.433	0.978	0.028	0.420	0.194	0.912
Block I plus levels of TIM CD68								
TIM CD68	0.011	0.573	0.373	0.881	0.005	0.698	0.545	0.895
Block I plus levels of TIL Foxp3								
TIL Foxp3	0.008	0.556	0.360	0.857	0.017	0.694	0.514	0.936
Block I plus level of desmoplasia								
Desmoplasia	0.084	1.202	0.976	1.480	0.408	1.295	0.702	2.391
Block I plus inflammation								
Inflammation	0.060	1.268	0.990	1.624	0.856	1.060	0.565	1.991
Block I plus level of nuclear polymorphism								
Nuclear polymorphism	0.950	1.009	0.764	1.333	0.363	0.722	0.358	1.457
Block I plus level of inflammation								
Invasion	0.484	1.091	0.855	1.391	0.603	0.853	0.469	1.552
RR: Relative risk.								
CI: Confidence interval.								
Abbreviations otherwise as in Table 1 and Table 2.								

## Data Availability

The data from this study are not allowed to be shared with anyone due to national legal regulations.
